# Genetic Diversity and Lack of Artemisinin Selection Signature on the *Plasmodium falciparum* ATP6 in the Greater Mekong Subregion

**DOI:** 10.1371/journal.pone.0059192

**Published:** 2013-03-26

**Authors:** Miao Miao, Zenglei Wang, Zhaoqing Yang, Lili Yuan, Daniel M. Parker, Chaturong Putaporntip, Somchai Jongwutiwes, Phonepadith Xangsayarath, Tiengkham Pongvongsa, Hazuhiko Moji, Trinh Dinh Tuong, Tomoko Abe, Shusuke Nakazawa, Myat Phone Kyaw, Guiyun Yan, Jeeraphat Sirichaisinthop, Jetsumon Sattabongkot, Jianbing Mu, Xin-zhuan Su, Osamu Kaneko, Liwang Cui

**Affiliations:** 1 Department of Entomology, Pennsylvania State University, University Park, Pennsylvania, United States of America; 2 Parasitology Department, Kunming Medical College, Kunming, Yunnan, China; 3 Molecular Biology of Malaria and Opportunistic Parasites Research Unit, Department of Parasitology, Chulalongkorn University, Bangkok, Thailand; 4 Department of Protozoology, Institute of Tropical Medicine (NEKKEN) and the Global Center of Excellence program, Nagasaki University, Japan; 5 Station of Malariology, Parasitology and Entomology, North Phonesavang Village, Kaysone District, Savannakhet Province, Laos; 6 Research Institute for Humanity and Nature, Kyoto, Japan; 7 Department of Epidemiology, National Institute of Malariology, Parasitology, and Entomology, Hanoi, Vietnam; 8 Parasitology Research Division, Department of Medical Research-Lower Myanmar, Yangon, Myanmar; 9 Program in Public Health, University of California Irvine, Irvine, California, United States of America; 10 Vector-borne Disease Training Center, Saraburi, Thailand; 11 Faculty of Tropical Medicine, Mahidol University, Bangkok; 12 Laboratory of Malaria and Vector Research, National Institute of Allergy and Infectious Diseases, National Institutes of Health, Bethesda, Maryland, United States of America; Instituto de Higiene e Medicina Tropical, Portugal

## Abstract

The recent detection of clinical Artemisinin (ART) resistance manifested as delayed parasite clearance in the Cambodia-Thailand border area raises a serious concern. The mechanism of ART resistance is not clear; but the *P. falciparum* sarco/endoplasmic reticulum Ca^2+^-ATPase (PfSERCA or PfATP6) has been speculated to be the target of ARTs and thus a potential marker for ART resistance. Here we amplified and sequenced *pfatp6* gene (∼3.6 Kb) in 213 samples collected after 2005 from the Greater Mekong Subregion, where ART drugs have been used extensively in the past. A total of 24 single nucleotide polymorphisms (SNPs), including 8 newly found in this study and 13 nonsynonymous, were identified. However, these mutations were either uncommon or also present in other geographical regions with limited ART use. None of the mutations were suggestive of directional selection by ARTs. We further analyzed *pfatp6* from a worldwide collection of 862 *P. falciparum* isolates in 19 populations from Asia, Africa, South America and Oceania, which include samples from regions prior to and after deployments ART drugs. A total of 71 SNPs were identified, resulting in 106 nucleotide haplotypes. Similarly, many of the mutations were continent-specific and present at frequencies below 5%. The most predominant and perhaps the ancestral haplotype occurred in 441 samples and was present in 16 populations from Asia, Africa, and Oceania. The 3D7 haplotype found in 54 samples was the second most common haplotype and present in nine populations from all four continents. Assessment of the selection strength on *pfatp6* in the 19 parasite populations found that *pfatp6* in most of these populations was under purifying selection with an average *d_N_*/*d_S_* ratio of 0.333. Molecular evolution analyses did not detect significant departures from neutrality in *pfatp6* for most populations, challenging the suitability of this gene as a marker for monitoring ART resistance.

## Introduction

**Figure 1:**
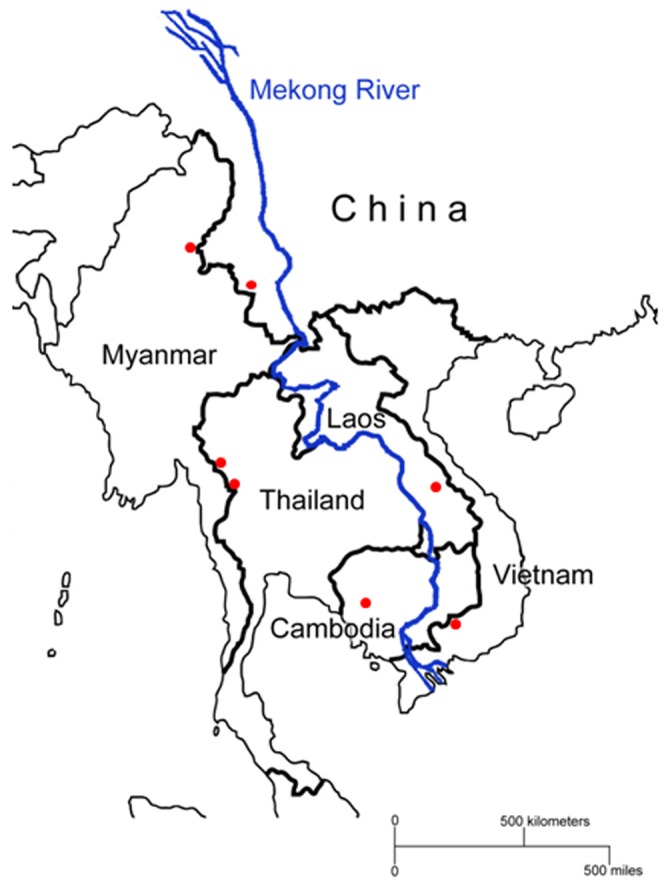
Map of the GMS. Origins of parasite samples are marked as red dots. The Mekong River is shown in blue.

The malignant malaria parasite *Plasmodium falciparum*, which still claims almost one million deaths per year [1], has exhibited an extraordinary ability to develop resistance to antimalarial drugs. To deal with the escalating problem of multidrug resistance (MDR), the World Health Organization (WHO) has advocated artemisinin (ART)-based combination therapies (ACTs) as the first-line treatment for uncomplicated falciparum malaria [2], a policy that has been adopted by most malaria-endemic countries [3]. Following the introduction of ACTs, an impressive reduction in malaria incidence, even in the malaria heartland, has brought renewed promises of malaria elimination/eradication [4], [5]. Given its central role in the contemporary agenda of malaria control and elimination, the recent reports of delayed clearance after ACT treatment in western Cambodia is a significant concern [6]. Careful clinical investigations have confirmed that ART resistance was manifested as prolonged parasite clearance as well as decreased *in vitro* susceptibility to ARTs [7]–[9]. Alarmingly, the delayed parasite clearance phenomenon has also been observed in other regions such as the Thai-Myanmar border area [10]. The Greater Mekong Subregion (GMS), bound by the Mekong River, includes Cambodia, China’s Yunnan Province, Laos, Myanmar, Thailand, and Vietnam (Figure 1). Historically, the GMS has been an epicenter of drug resistance, where chloroquine- and pyrimethamine-resistant parasites first emerged and spread to Africa [11], [12]. To prevent an analogous spread of ART resistance from happening again, containment and monitoring measures should be deployed across the GMS regions [13].

The exact mode of antimalarial action of ART drugs is still under debate [14]. It has been proposed that ARTs target the sarcoplasmic/endoplasmic reticulum Ca^2+^ ATPase (PfSERCA or PfATP6), since the heterologously expressed PfATP6 in *Xenopus laevis* oocytes can be inhibited by ARTs as well as thapsigargin, a sesquiterpene lactone that specifically inhibits mammalian SERCAs [15]. Furthermore, replacement of leucine at codon 263 with glutamate, which is predicted to be located in the ART-binding site, causes abrogation of inhibition of PfATP6 [16]. However, introduction of the L263E mutation in *P. falciparum* through allelic exchange did not result in similarly dramatic changes in parasite’s susceptibility to ARTs [17]. So far, mutations at position 263 have not been detected in field isolates from regions with suspected ART resistance. When comparing sensitivities to ARTs in field isolates from Cambodia, French Guiana, and Senegal, Jambou *et al*. found a S769N substitution in a limited number of parasite field isolates from French Guiana, which was associated with significantly reduced *in vitro* susceptibility to artemether [18]. Additionally, two mutations at codons 431 and 623 were also found to be associated with higher IC_50_s to artesunate (ATS) in three isolates from Senegal [18], [19]. Despite numerous genotyping studies, the S769N substitution has not been detected elsewhere [19]–[27] except for another isolate from Africa [28]. However, this isolate carrying the S769N mutation was sensitive to dihydroartemisinin (DHA) and artemether (ATM) from *in vitro* analysis [28]. Recently, contrary to the reported scarcity of S769N mutations in world *P. falciparum* populations, the S769N mutation was found at high frequency in isolates in travelers returning from a range of African countries [29], [30]. Surprisingly, these authors also identified the A623E/S769N double mutations in 39% of the isolates tested, which were associated with increased resistance to ATM [30]. However, the reliability of these data was subsequently questioned and the primers used to sequence *pfatp6* appeared inadequate [31]. Our recent study using allelic replacement did not detect the influence of the S769N mutation on parasite’s *in vitro* sensitivity to DHA, ATS or ATM [32]. Therefore, the role of PfATP6 in modulating ART resistance in *P. falciparum* needs further examination.

A recent large-scale analysis of *pfatp6* gene from global *P. falciparum* populations prior to the deployment of ACTs has provided baseline information about the spontaneous mutations in *pfatp6* [22]. Whereas sequence variations are characterized by the abundance of geographic region-specific mutations, the majority of these mutations are observed at low frequencies. Since the GMS has the longest history of ART use, we reason that if *pfatp6* were the molecular target of ARTs, we would expect to find potential signatures of selection resulted from extensive deployment of this family of drugs in the GMS. To address this question, we undertook a comprehensive analysis of the diversity of *pfatp6* from isolates obtained from the GMS. We examined polymorphisms in this gene in different geographic locations to infer whether the observed level of diversity is greater between populations than within populations. Based on the genetic diversity of *pfatp6*, we tested the underlying selective forces that acted on this gene. Our study did not detect apparent selection of this gene in worldwide parasite populations, suggesting that *pfatp6* is not a suitable marker for monitoring ART resistance.

## Materials and Methods

### Sample Collection

In recent years, the malaria situation in the GMS has been improved significantly with most of countries becoming malaria-hypoendemic except Myanmar [33]. However, malaria distribution is highly heterogeneous and concentrated along international borders, where most of the parasite samples were collected (Figure 1). Clinical samples of *P. falciparum* were collected after 2005 from patients presenting with uncomplicated falciparum malaria at local clinics in these GMS countries. *P. falciparum* infections were confirmed by light microscopy on Giemsa-stained thin and thick blood smears. The geographic locations of the parasite populations are depicted in Figure 1, including Cambodia (Pursat), China (Gengma county, Yunnan Province), Laos (Sepone, Savannakhet Province), Myanmar (Kachin State), Thailand (Phop Phra District and Tha Song Yang District, Tak Province), and Vietnam (Phu Thuan Village, Phu Rieng Commune, Phuoc Long District, Binh Phuoc Province). Finger-prick blood samples were collected after written informed consent and received ethical clearance from the local ethical committees of the respective countries. Blood samples were absorbed onto Whatman filter papers (Whatman Inc., Florham Park, NJ, USA) and stored at −20°C until DNA extraction. Use of the samples for the current study was approved by the Institutional Review Board of Pennsylvania State University.

### DNA Extraction, PCR and Sequencing

DNA was extracted from filter papers using QIAamp DNA Mini Kit (Qiagen, Valencia, CA). Parasites were genotyped at three polymorphic loci to exclude multiclonal infections [34]. The primer sequences for the amplification of *pfatp6* are listed elsewhere, which do not cover the introns [19], [35]. High-fidelity PCR was carried out using Advantage DNA Polymerase Mix (Clontech, Mountain View, CA), which has efficient 3′ 5′ exonuclease proof-reading activity. PCR reactions were performed in 25 µl with 2 µl of DNA template, 0.2 µM of each primer, 2.5 µl 10 × PCR buffer, 2.5 units of DNA polymerase mix, and 0.2 mM dNTP mix. Reaction conditions consisted of an initial denaturation at 94°C for 5 min followed by 35 cycles of 94°C for 20 sec, 60°C for 30 sec and 72°C for 1 min, and a final extension step of for 10 min at 72°C. All PCR products were purified with PrepEase Gel Extraction Kit (United States Biochemicals, Cleveland, OH) or ExoSap_IT (United States Biochemicals) and sequenced in both directions using the BigDye 3.1 Cycle Sequencing Kit (ABI). Low-quality sequences were excluded from analysis and re-sequenced using additional primers to rule out sequencing error.

### Sequences Alignment

PHRED [36], [37], PHRAP (www.phrap.org), and CONSED [38] were used to call bases, assemble sequences into contigs, and view assemblies, respectively. Sequences were aligned in Bioedit v.7.0.9.0 (http://www.mbio.ncsu.edu/bioedit/bioedit.html) using the ClustalW algorithm [39], using the 3D7 *pfatp6* sequence (PF3D7_0106300) as the reference (www.plasmodb.org). The nucleotide sequence data reported in this paper are available in the GenBank database under the accession numbers (JN983240–JN983286, JN983288–JN983290, and KC576980–KC577142). In addition, we have retrieved 649 additional *pfatp6* sequences from the GenBank for comparison.

### Tests of DNA Diversity, Neutrality and Demographic Analyses

Sequences were imported into DNASP v5.10 [40] for analyses of nucleotide composition and genetic variation. The number of segregating sites (*S*), haplotype diversity (*h*), and nucleotide diversity (*π*) were estimated for each sampling site. To study the molecular evolution of *pfatp6*, we performed three tests of natural selection: 1) The rates of nonsynonymous (*d*N) and synonymous (*d*S) substitutions in *pfatp6* were estimated (http://www.hiv.lanl.gov). If positive selection has operated since the divergence of the alleles, the ratio of *d*N/*d*S will exceed one; conversely, when purifying selection is acting on the gene, the *d*N/*d*S ratios will be less than one. For *d*N/*d*S ratios, significance was determined by the two tailed *Z*-test (*P*<0.05) of selection using the Nei and Gojobori method [41] implemented in MEGA version 4.0 [42] with the Jukes and Cantor (JC) correction. A bootstrap method with 1000 pseudoreplicates was used to estimate variances. 2) Departure from selective neutrality was assessed using the McDonald and Kreitman (MK) test [43] on DNASP 5.10 [40]. For between-species comparisons, sequence of the *atp6* gene from *P. reichenowi* was used as outgroup. The neutrality index (*NI*) was calculated from the equation *NI* = (Pn/Ps)/(Fn/Fs), where Pn and Ps are the numbers of nonsynonymous and synonymous polymorphisms, respectively, whereas Fn and Fs are the nonsynonymous and synonymous fixed differences, respectively. Fisher’s exact test was used for the significance of the MK test. 3) Tajima’s *D* [44] and Fu and Li’s D* and F* tests [45] were done in Arlequin 3.11 [46]. Tajima’s *D* test was based on the difference between *θ* (Watterson’s population nucleotide diversity parameter) and *π* (average pairwise nucleotide diversity). Under the neutral model, the expectation of *θ* should be the same as that of *π*, but under balancing selection, a positive *D* value is expected [44]. Fu and Li’s D* and F* test takes into account the difference between the observed number of singleton nucleotide polymorphisms and the number expected under neutrality from *S* (the total number of segregating sites) and *θ* [45].

### Haplotype Network Reconstruction

Aligned sequences were imported into Bionumerics software version 6.1 (Applied-Maths, St.-Martens-Latem, Belgium) for estimating gene genealogies, and minimum spanning trees (MSTs) were constructed using the method of Templeton et al. [47]. MSTs were used to group subtyping data and to visualize the relationship between large numbers of isolates. Outgroup probabilities were estimates of the relative haplotype age based on the haplotype frequency and the number of connections to other haplotypes in the network [48].

### Genetic Differences between Populations

Genetic population structure was investigated using hierarchical analysis of molecular variance (AMOVA), using both *F*_ST_ (based on haplotype frequencies) and *Φ*_ST_ (based on genetic distances between haplotypes) contained within Arlequin version 3.1. Whereas an *F*_ST_ and *Φ*_ST_ score value of 0 between two populations would indicate that they are completely undifferentiated, a score of 1 would indicate that every observable genetic difference between individuals of the two populations can be used to distinguish between the populations. The significance of the two fixation indices was tested through 10,000 permutations for each pairwise comparison. Because the TVM+I+G model given by MODELTEST [49] is not available in Arlequin, genetic distances between haplotypes were corrected for multiple hits using the Tamura and Nei model of nucleotide substitution [50] with a gamma (Г = 0.32) correction for heterogeneity of mutation rates.

To detect clustering of the haplotypes within the GMS populations, a metric multidimensional scaling (MDS) analysis was conducted. A distance matrix (identity by state) was constructed used to calculate three eigenvectors or principal components (PCs). The three PCs were then mapped onto two dimensional graphs for easy visualization. The data were analyzed using the “bios2mds” package for R [51].

## Results

### Genetic Diversity of *pfatp6* in Parasites Collected from the GMS and the World

We sequenced the entire coding region of *pfatp6* gene (3687 bp excluding introns, encoding 1228 amino acids) in 213 *P. falciparum* clinical samples from six countries of the GMS (Figure 1). Together with 81 samples obtained from Thailand in an earlier study [52], a total of 294 complete *pfatp6* sequences obtained from the GMS were included in the analysis. From the GMS dataset, we identified 24 single nucleotide polymorphisms (SNPs) (indels not considered), of which 13 were nonsynonymous substitutions. All polymorphic sites were dimorphic with two alternative nucleotides. Eight SNPs at positions **73**, 231, **250**, **676**, 924, **1945**, **2128**, and 2340 were novel in the GMS dataset, among which five (in bold) were nonsynonymous. SERCA has three conserved domains: cytoplasmic domain, transmembrane gate and luminal loops [53]. Within the *pfatp6* molecule, 19 SNPs were located in the cytoplasmic domain, whereas the predicted transmembrane and luminal domains had only one and four SNPs, respectively (Table 1). A sliding window analysis of nucleotide diversity in the GMS samples with a window of 100 bp and a step size of 25 bp revealed that the maximum diversity was located between nucleotide positions 3000 to 3500 (Figure S1). Interestingly, six of the 11 synonymous SNPs were clustered in an 862 bp region towards the 3′ end of *pfatp6*. For the 13 nonsynonymous mutations, three were located in the luminal regions and 10 in the cytoplasmic region. It is noteworthy that none of nonsynonymous SNPs were found in the putative ART-binding domains (256–269, 985–991) [53]. Moreover, the L263E and S769N substitutions proposed to be important for ART resistance [16], [18] were not found in parasite isolates from the GMS. The E431K substitution, which was associated with increased artesunate IC50s in Senegal [18], was found in 1 of 116 (0.86%) samples collected in Myanmar and 2 of 13 (15.4%) in Vietnam. The I89T mutation detected in earlier Thai samples [8] was also found in most GMS parasite populations, and reached relatively high prevalence in samples from the China-Myanmar border region.

**Table 1:** List of the *pfatp6* mutations detected in samples from the GMS.

Nucleotideposition	Codon	Wild-typecodon	Mutant codon	Amino Acid	Location in domain	Number of Samples						
						China (N = 26)	Laos (N = 8)	Myanmar (N = 116)	Cambodia (N = 29)	Vietnam (N = 13)	Thailand^1^ (N = 21)	Thailand^2^ (N = 81)
**73**	25	GAT	AAT	**D-N**	cyto						2	
231	77	TTC	TTT	F**-**F	luminal	1						
250	84	ATG	ATA	**M-I**	luminal					1		
**266**	89	ATA	ACA	**I-T**	luminal	7		10	1		1	6
676	226	ATA	GTA	**I-V**	cyto			1				
924	308	GCT	GCC	A**-**A	TM					1		
1086	362	ACC	ACT	T**-**T	cyto			1				
1204	402	TTA	GTA	**L-V**	cyto							1
**1291**	431	AGA	AAA	**R-K**	cyto			1		2		
**1313**	438	GCT	GAT	**A-D**	cyto	8		6			2	2
**1394**	465	AAT	AGT	**N-S**	cyto			2	1	2		4
**1449**	483	AAT	AAC	N**-**N	cyto			3				
**1707**	569	AAT	AAA	**N-K**	cyto					1		4
1890	630	GCT	GCC	A**-**A	cyto							1
1945	649	AAA	GAA	**K-E**	cyto			1				
**2049**	683	AAT	AAA	**N-K**	cyto		1	4				6
2128	710	GAA	AAA	**E-K**	cyto			1				
**2268**	756	AGA	AGG	R**-**R	cyto	9		5				1
2340	780	GAT	GAC	D**-**D	cyto			1				
**2694**	898	ATT	ATA	I**-**I	cyto		1	19			5	4
2877	959	ATT	ATA	I**-**I	cyto				1			
**3090**	1030	AAG	AAA	K**-**K	cyto			3	9	1	1	1
**3093**	1031	TGC	TGT	C**-**C	cyto	1	1	7	3	2	2	12
3562	1187	GCA	ACA	**A-T**	luminal	1						

All nucleotide, codon and corresponding amino acid positions are adjusted to correspond to positions in *pfatp6* sequence of 3D7 (*PF3D7_0106300*). Location of the amino acids in the protein domain: cyto – cytoplasmic; TM – transmembrane. Nucleotide positions highlighted in bold are evolutionarily informative.

1 Sequences obtained in this study,

2 Sequences from Tanabe et al. (2004).

Most SNPs found in the GMS were relatively rare; the highest prevalence of synonymous and nonsynonymous mutations was 9.8% and 8.5% at nucleotides 2694 and 266 (I89T), respectively (**Table 1**). SNPs at 12 positions 231, 250, 676, 924, 1086, 1204, 1890, 1945, 2128, 2340, 2877, and 3562 were singletons. Similarly, except for the SNP at position 73, each novel SNP was only found in a single parasite isolate. In addition, the prevalence of individual SNPs also differed geographically and appeared to be population-specific. Whereas SNPs at positions 266, 1313, and 2268 had similar levels of prevalence in the Chinese samples, the SNP at position 2694 was the most prevalent in the samples collected from the adjacent Myanmar site. In the Cambodian samples, mutation at position 3090 was the most predominant. Interestingly, in the earlier Thai samples mutation at position 3093 was also the predominant, whereas mutation at position 2694 was the most prevalent in the later Thai samples.

In parasite samples from the GMS, the haplotype diversity (*h*) was relatively high with an average of 0.654 (Table 2). The parasite population from Laos had the lowest haplotype diversity of 0.464, whereas the haplotype diversity of the remaining five populations was clustered in a small range of 0.614–0.731. Consistent with the rarity of SNPs, nucleotide diversity (*π*) of *pfatp6* was also low, ranging from 0.00020 to 0.00043, with the population from China showing the highest π value (Table 1). Overall, θs, the standardized number of polymorphic sites per site, was higher than θ_π_, which further indicates that the majority of alleles in the GMS were infrequent.

**Table 2:** Genetic diversity of *pfatp6* sequences obtained from malaria-endemic countries of the GMS*.

Sample sites	Time of collection	NoSamples	No. of haplotypes	No. of unique haplotypes	*S*	*h*	*π*	*k*
China	2005	26	8	3	6	0.695±0.091	0.00043±0.00007	1.588
Laos	2010	8	3	0	3	0.464±0.200	0.00020±0.00010	0.750
Myanmar	2007–2009	116	19	6	15	0.614±0.049	0.00027±0.00003	0.991
Cambodia	After 2005	29	5	1	5	0.645±0.065	0.00023±0.00004	0.842
Vietnam	2006	13	7	2	7	0.731±0.133	0.00040±0.00011	1.462
Thailand^1^	2007–2008	21	8	3	6	0.724±0.101	0.00030±0.00006	1.114
Thailand^2^	1995	81	12	1	11	0.643±0.053	0.00028±0.00004	1.016
**Total**		294						

* Genetic diversity indices: *S*, segregating sites; *h*, haplotype diversity; *π*, nucleotide diversity; *k*, average number of pairwise differences.

1 Sequences obtained in this study.

2 Sequences from Tanabe et al. (2004).

By the time of this study, 649 additional *pfatp6* sequences were available in GenBank, which represent parasites from 19 populations from Asia, Africa, South America and Oceania. The 68 *pfatp6* sequences recently obtained in southern Ghanaian samples were partial sequences and were excluded in our analysis [54]. Comparison of all *pfatp6* sequences identified a total of 71 SNPs, including 30 synonymous and 41 non-synonymous. Ten SNPs are located within the predicted transmembrane domain (six nonsynonymous), whereas eight are located within the luminal domain (two nonsynonymous). When all samples were grouped by continent, the average haplotype diversity of the populations from Africa (0.922) was the highest, followed by South America, and Asia (Table S1). Populations from the Pacific Islands had the lowest haplotype diversity (0.290), suggesting relatively few *pfatp6* haplotypes were circulating in the sampling areas. The rank order of nucleotide diversity (*π*) was similar to that of haplotype diversity>Africa (0.00061)>Asia (0.00034) >Pacific Islands (0.00018) with the exception of South America (0.00083) (Table S1).

### Natural Selection on *pfatp6*

To detect signatures of natural selection on the *pfatp6* gene, we first compared the ratio of nonsynonymous to synonymous substitutions (d*N*/d*S*). The results showed that d*N*/d*S* ratio for the GMS samples or the entire sample set was below one, suggesting purifying selection. *Z*-test revealed that either within continent or the entire sample collection there was no significant difference between d*N* and d*S*, suggesting that d*N*/d*S* ratio did not significantly deviate from the neutral expectation (Table 3). To corroborate this conclusion, three additional neutrality tests – the MK test, Tajima’s *D* test and Fu & Li’s *D* and *F* tests were performed, and the results were highly agreeable between the tests (Table 3). Tajima’s *D* test for individual parasite populations from the GMS only detected marginally significant departures from neutrality in two populations (Table 3). In agreement with the access of rare singletons, Tajima’s D, Fu and Li’s D* and F* were negative in most populations. Similarly, the MK test performed for each population using SNPs in *P. falciparum* and its divergence from *P. reichenow*i detected an excess of synonymous fixed differences between the two species. Seven populations showed significant departures from neutrality, and they were found in certain populations from all continents except the Pacific Islands. However, most of the tests were insignificant after Bonferroni corrections. In addition, the neutrality indexes (NI) were well above one [55], further indicating that purifying selection might have acted on this gene (Table 3). These analyses indicated that *pfatp6* gene in most parasite populations did not deviate significantly from neutrality and there was significant heterogeneity between individual populations, suggesting population differentiation.

**Table 3:** Tests of neutrality for *pfatp6* gene (dN/dS, Tajima’s *D*, Fu and Li’s F* and D* statistics, and McDonald-Kreitman test) among *P. falciparum* populations from different regions of the world.

Samples	*d*N*/d*S ratio		Tajima’s *D*		Fu & Li’s D*		Fu & Li’s F*				McDonald-Kreitman test										
	*d*N/*d*S	*P*	*D*	*P*	*D*	*P*	*F*	*P*		*Ps*	*Pn*	*Ds*				*Dn*	*NI*		*P*		
China	0.326	0.446	0.0289	0.5440	−1.023	NS	−0.831	NS		3	3		51		15			3.400		0.161	
Laos	0.213	0.206	−1.4475	0.0660	−1.565	NS	−1.686	NS		2	1		51		15			1.700		1.000	
Myanmar	0.267	0.131	−1.7595	**0.0130**	−1.762	NS	−2.102	NS		7	8		51		15			3.886		**0.027**	
Cambodia	0.487	0.162	−0.9313	0.1900	−1.463	NS	−1.518	NS		3	2		51		15			2.267		0.587	
Vietnam	0.302	0.308	−1.3353	0.0860	−0.854	NS	−1.115	NS		3	4		51		15			4.533		0.070	
Thailand^1^	0.218	0.190	−1.0423	0.1670	−0.177	NS	−0.488	NS		3	3		51		15			3.400		0.161	
Thailand^2^	0.243	0.289	−1.4566	**0.0470**	1.120	NS	−1.467	NS		5	6		51		15			4.080		0.061	
Philippines	0.236	0.445	0.0136	0.5230	−0.113	NS	−0.086	NS		2	2		51		15			3.400		0.246	
Bangladesh	0.256	0.345	−1.2326	0.0890	−1.027	NS	−1.280	NS		2	5		51		15			8.500		**0.014**	
Iran	0.214	0.185	−0.1005	0.4970	−0.131	NS	−0.143	NS		6	5		50		15			2.778		0.145	
GMS	0.294	0.076	NA		NA		NA			11	13		51		15			4.018		**0.009**	
Asia	0.294	0.086	NA		NA		NA			15	16		50		15			3.556		**0.009**	
PNG	0.235	0.166	−1.6214	**0.0220**	−0.314	NS	−0.895	NS		4	4		51		15			3.400		0.192	
Solomon Islands	0.243	0.227	−1.2945	0.1010	1.002	NS	0.346	NS		2	2		51		15			3.400		0.246	
Vanuatu	0.487	0.644	0.8387	0.8090	0.846	NS	0.988	NS		1	2		51		15			6.800		0.148	
Pacific Islands	0.438	0.199	NA		NA		NA			6	5		51		15			2.833		0.141	
Ghana	0.239	0.253	−1.5908	**0.0420**	−2.725	*	−2.776	*		6	7		49		15			3.811		**0.042**	
Sudan	0.000	0.154	0.0000	0.9370	NA	NA	NA	NA		0	2		51		15			NA		NA	
Tanzania	0.230	0.156	−1.9190	**0.0100**	−2.455	*	−2.714	*		12	19		49		15			5.172		**0.001**	
Malawi	0.284	0.384	−1.2165	0.1090	−1.602	NS	−1.739	NS		4	10		51		15			8.500		**0.001**	
Madagascar	0.274	0.326	−0.1686	0.4740	0.477	NS	0.339	NS		4	6		51		15			5.100		**0.023**	
Africa	0.316	0.208	NA		NA		NA			17	27		47		15			4.976		**0.000**	
Brazil	0.419	0.532	0.6290	0.7760	0.729	NS	0.818	NS		3	6		51		15			6.800		**0.012**	
Venezuela	0.398	0.241	−1.2447	0.1300	−1.127	NS	−1.294	NS		2	2		51		16			3.188		0.265	
South America	0.474	0.472	NA		NA		NA			3	7		51		15			7.933		**0.005**	
Worldwide	0.333	0.125	NA		NA		NA			28	41	46		15			4.490			**0.000**	

**Fu & Li's** D* **and** F* **:** NS – not significant; NA – not available; * – significant, P<0.05;

**McDonald-Kreitman test:** Ps, Pn – synonymous and nonsynonymous polymorphic sites within *P. falciparum*; Ds, Dn – synonymous and nonsynonymous fixed nucleotide differences between species; *P* – value of Fisher’s Exact tests; NI – neutrality index. Significant *P* values are highlighted in bold.

1 Sequences obtained in this study,

2 Sequences from Tanabe et al. (2004).

### Haplotype Distribution

**Figure 2:**
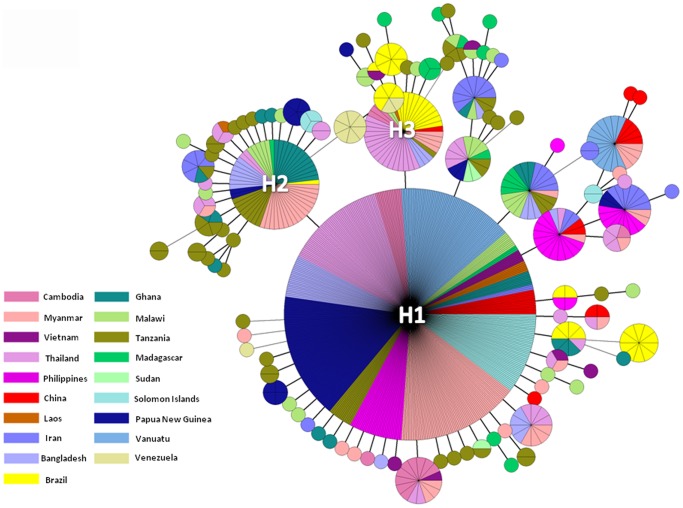
Unrooted minimum spanning tree network showing genetic relationship among *pfatp6* gene haplotypes for the worldwide populations of *P. falciparum*. Each distinct haplotype is represented by a pie with the size proportional to its frequency. Mutational steps are represented by lines connecting the pies, whereas the country origins of the parasite haplotypes are shown by different colors.

Analysis of 862 available *pfatp6* sequences from the world identified 106 haplotypes based on 71 polymorphic sites. The relationships among these haplotypes were inferred by the MST method, which revealed that three haplotypes were in high frequencies and common for most parasite populations studied (Figure 2). Haplotype H1 was considered the ancestral haplotype as it connected unambiguously to the outgroup – *P. reichenowi atp6* gene (data not shown). It was also the most common haplotype occurring in approximately half of all individuals sampled (444/862, 51.5%) and had the widest geographic distribution across 16 populations (with the exception of samples from Brazil, Venezuela and Sudan). H1 is connected to several low-frequency haplotypes, generating a star-like genealogy, which is indicative of a possible past range expansion. The reference sequence from 3D7, referred hitherto as haplotype H2, differed from H1 by a T to C transition at position 3236, and was the second most common haplotype in the entire population (56/862, 6.5%). Although H2 was restricted to nine populations, it was present in all continents analyzed. The third most represented haplotype (H3) (44/862, 5.1%, transition at position 2694) was also found in nine populations in Asia, Africa and South America. All remaining haplotypes, including 65 singleton (unshared) haplotypes, were found in the remaining 318 samples. Derivatives with lower frequencies were connected to the dominant haplotype by up to eight mutational steps (Figure 2). No distinct segregation was found among four continents (Figure 2, S2). The haplotype networks of individual continents showed similar patterns of haplotype distribution (Figure S2). Furthermore, we generated a worldwide structure of haplotype frequencies at 41 nonsynonymous/amino acid sites (Figure S3). The topology of the network showed that the haplotype AH1, representing the nucleotide polymorphisms in H1, H2 and H3 of the nucleotide network, was found in the majority of the global samples (Figure 2, S3).

**Figure 3:**
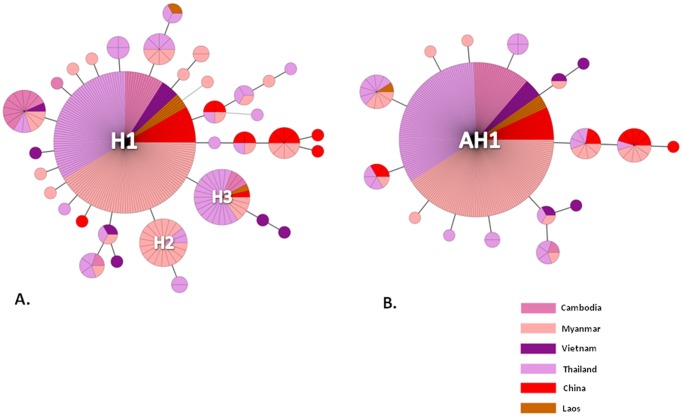
Unrooted minimum spanning tree network showing genetic relationship among *pfatp6* gene haplotypes for *P. falciparum* populations collected from the GMS. A) Data based on *pfatp6* gene haplotypes; B) Data based on PfATP6 protein haplotypes.

Within the GMS, a total of 34 haplotypes were detected ranging from 3 to 19 in each of the individual population (Table 2). MST analysis of the 34 haplotypes generated a radiating network, confirming the genetic inter-relatedness of these haplotypes (Figure 3A). H1 remained as the central haplotype in the network (169/294, 57.5%), and was present in samples from all six GMS countries. Haplotype H3 was the second dominant haplotype and present in five populations. However, H2 was only found in populations from Myanmar and Thailand. In different continents, the proportions of the three major haplotypes (H1, H2 and H3) also varied. In South America, H1 was absent, whereas H3 was the predominant haplotype. In Pacific Islands and Africa, haplotype H3 was absent (Figure S2). Amino acid haplotype frequencies were similar between the worldwide and GMS populations (Figure 3B, S3).

### Differentiation within and between Populations

We used AMOVA to evaluate the degree of population subdivision. We calculated the *F*_ST_ and *Φ*_ST_ values for pairwise comparisons among the 20 global populations. Results obtained from the two tests were comparable, albeit a higher level of statistical significance was obtained when the sequence information was used (*Φ*_ST_) (Table 4). Comparisons with full sequence data among all populations generated *F*_ST_ values ranging from 0 to 0.845, indicating different levels of population differentiations. More than half of the comparisons generated *F*_ST_ values of >0.15, suggesting of significant population subdivision of parasite populations. Thirty seven pairwise comparisons had small *F*_ST_ values (<0.049), suggesting little population differentiation, whereas 54 comparisons had *F*_ST_ values of 0.05–0.148, suggesting of low – intermediate levels of population differentiation. The highest level of subdivision was observed when comparing the parasite population from Venezuela with any other populations (*F*_ST_ values >0.469) (Table 4). Of the six GMS populations studied, significant population subdivision was only evident between the population from China and other GMS populations with *F*_ST_ ranging from 0.144 to 0.228 (Table 4). Population differentiation among other GMS populations was from low to modest levels (*F*_ST_ values <0.101).

**Table 4:** Population differentiation indices comparing 20 worldwide *P. falciparum* populations.

	1	2	3	4	5	6	7	8	9	10	11	12	13	14	15	16	17	18	19	20
1. China	–	0.275**	0.206**	0.352**	0.312**	0.251**	0.248**	0.212**	0.303**	0.521**	0.280**	0.242**	0.033*	0.563**	0.619**	0.562**	0.454**	0.593**	0.940**	2.908**
2. Laos	0.144*	–	–0.019	0.070	0.000	–0.022	–0.030	0.162**	–0.042	0.352**	–0.012	–0.008	0.087**	0.144**	0.334**	0.154**	0.082	0.230**	0.521**	2.358**
3. Myanmar	0.163*	–0.035	–	0.105**	0.055**	–0.004	0.034**	0.127**	0.009	0.336**	0.018**	0.015**	0.062**	0.147**	0.373**	0.170**	0.128**	0.288**	0.657**	2.365**
4. Cambodia	0.228*	0.074	0.097*	–	0.045	0.106**	0.089**	0.232**	0.119**	0.470**	0.103**	0.100**	0.171**	0.371**	0.430**	0.364**	0.245**	0.376**	0.674**	2.692**
5. Vietnam	0.166*	–0.015	0.064*	0.053	–	0.064	0.004	0.195**	0.024	0.237**	0.037**	0.045**	0.126**	0.264**	0.270**	0.205**	0.065	0.126	0.549**	2.615**
6. Thailand^1^	0.153*	–0.037	–0.002	0.101*	0.053	–	0.038**	0.188**	0.009	0.363**	0.031**	0.029**	0.099**	0.092**	0.396**	0.123**	0.100**	0.269**	0.591**	2.229**
7. Thailand^2^	0.181*	–0.047	0.033*	0.082*	0.017	0.037*	–	0.153**	0.026**	0.336**	0.039**	0.040**	0.096**	0.256**	0.316**	0.236**	0.119**	0.249**	0.508**	2.519**
8. Philippines	0.164*	0.150*	0.116*	0.209*	0.175*	0.167*	0.136*	–	0.179**	0.281**	0.148**	0.138**	0.115**	0.434**	0.495**	0.430**	0.332**	0.451**	0.855**	2.797**
9. Bangladesh	0.213*	–0.059	0.008	0.120*	0.038	0.013	0.025*	0.168*	–	0.288**	0.020**	0.024**	0.117**	0.127**	0.342**	0.126**	0.072**	0.193**	0.613**	2.359**
10. Iran	0.192*	0.102*	0.206*	0.205*	0.083*	0.145*	0.190*	0.156*	0.152*	–	0.367**	0.389**	0.409**	0.408**	0.414	0.266**	0.154**	0.107	0.852**	2.748**
11. PNG	0.274*	0.000	0.021*	0.146*	0.093*	0.059*	0.048*	0.181*	0.032*	0.255*	–	0.006	0.090**	0.203**	0.299**	0.206**	0.132**	0.285**	0.676**	2.497**
12. Solomon	0.244*	0.026	0.014	0.159*	0.114*	0.063*	0.047*	0.177*	0.039*	0.238*	0.011	–	0.066**	0.225**	0.340**	0.237**	0.159**	0.310**	0.690**	2.534**
13. Vanuatu	0.039	0.084	0.061*	0.164*	0.131*	0.100*	0.091*	0.115*	0.117*	0.233*	0.113*	0.085*	–	0.367**	0.421**	0.369**	0.268**	0.410**	0.786**	2.723**
14. Ghana	0.264*	0.068	0.119*	0.229*	0.146*	0.059*	0.180*	0.273*	0.098*	0.166*	0.200*	0.209*	0.254*	–	0.506**	0.023	0.106**	0.275**	0.735**	1.924**
15. Sudan	0.269*	0.313*	0.300*	0.366*	0.148	0.274*	0.263*	0.384*	0.315*	0.079	0.430*	0.541*	0.357	0.234*	–	0.287	0.164	0.306	1.034**	2.973**
16. Tanzania	0.197*	0.024	0.101*	0.147*	0.069*	0.044*	0.123*	0.192*	0.060*	0.096*	0.130*	0.127*	0.186*	0.008	0.039	–	0.021	0.119**	0.738**	2.107**
17. Malawi	0.194*	0.008	0.099*	0.135*	0.025	0.049*	0.086*	0.196*	0.049*	0.063*	0.126*	0.131*	0.180*	0.055*	0.023	0.008	–	0.021	0.539**	2.275**
18. Madagascar	0.225*	0.067	0.210*	0.199*	0.047	0.127*	0.176*	0.261*	0.134*	0.040	0.264*	0.258*	0.268*	0.130*	0.037	0.046*	0.013	–	0.577**	2.594**
19. Brazil	0.298*	0.157*	0.323*	0.263*	0.183*	0.216*	0.252*	0.347*	0.266*	0.248*	0.368*	0.339*	0.355*	0.260*	0.251*	0.227*	0.188*	0.181*	–	2.510**
20. Venezuela	0.670*	0.731*	0.704*	0.755*	0.676*	0.676*	0.713*	0.756*	0.725*	0.544*	0.811*	0.845*	0.754*	0.570*	0.746*	0.469*	0.547*	0.543*	0.519*	–

* significant at *P*<0.05 by the permutation test;

** significant *P* values after Bonferroni correction. *Φ*_ST_ and F_ST_ values are above and under the diagonal, respectively.

We next grouped the populations by continent and analyzed population structure between continents (Table S2). The *F*_ST_ values among Asia, Pacific Islands and Africa ranged from 0.017 to 0.137, suggesting of low to modest levels of population subdivision. However, the South American population differed substantially from populations from other continents. The high *Φ*_ST_ values obtained from comparing the South American population with populations from other continents (0.540–0.663) were highly significant even after Bonferroni corrections. At the continent level, parasite population from the GMS had a high degree of differentiation from the South American population (*F*_ST_ = 0.288, P<0.05). A moderate degree of differentiation was noticed for the GMS and Africa populations (*F*_ST_ = 0.101, P<0.05). In contrast, there was little genetic differentiation between the GMS and Pacific Island populations (*F*_ST_ = 0.015, P<0.05). Pairwise comparisons revealed that all continental groups were significantly different from each other, and the values remained significant even after Bonferroni correction.

To further support this finding, we performed MDS analysis of the GMS parasite populations. From the distance matrix, three PCs were identified with much of the variance (eigenvalue percentage) explained by PC1 (Figure S4). PCA plots showed little clustering of the parasite haplotypes by their origins within the GMS parasite populations (Figure S4). Consistently, a haplotype from China showed larger genetic distance from the rest of the GMS populations.

## Discussion

*SERCA* is an evolutionarily conserved protein and plays an important role in cellular homeostasis and calcium signaling functions [56], [57]. *P. falciparum* has only one SERCA gene, *pfatp6*, and it has been proposed as a specific target of ARTs [15]. The present study aimed to analyze the extent of genetic polymorphisms in *pfatp6* using a large collection of parasite isolates (n = 213) from the six GMS countries, where ART drugs have been used for the longest time. Our results confirmed the high level of genetic diversity (π = 0.00030) in *pfatp6* in the GMS, which is slightly lower than that observed for the entire sample set of 862 samples. The nucleotide diversity in *pfatp6* gene from the GMS was also somewhat lower than the recent estimate based on genome-wide SNPs [58]. As many as 13 nonsynonymous mutations were found in *pfatp6*, including five novel mutations. Among them, three were located in the luminal regions and 10 in the cytoplasmic region, but none was found in the putative ART-binding domain. Similar to previous reports, the majority of the SNPs were uncommon with frequencies below 5% and a substantial number of them were continent- and population-specific [19], [21], [22]. Of particular attention are the I89T and A438D mutations, which are relatively prevalent in China and Myanmar and also detected in Thailand. Although the I89T mutation was not detected in Africa and South America, it was prevalent in Iran and the Philippines. The A438D mutation was also highly prevalent in Oceania [22]. Furthermore, clinical studies conducted in Palin, Cambodia where delayed parasite clearance is evident, and in Wang Pha at the Thai-Myanmar border where ARTs are still very effective also detected the I89T mutation, but it was at similar frequencies at the two study sites and not associated with the resistance phenotype [8]. The E431K and A623E double mutant previously associated with reduced sensitivity to ART was not observed in our GMS samples [18], but the E431K mutation was found in 1 of 116 (0.86%) samples collected in Myanmar and 2 of 13 (15.4%) in Vietnam. The L263E and S769N mutations that were associated with increased resistance to ARTs were not detected in the GMS samples [16], [18]. Furthermore, the significance of these two mutations for ART resistance was not supported by genetic analysis where the mutant gene was introduced through allelic replacement [17], [32]. Taken together, this study analyzed genetic diversity of *pfatp6* in a large global collection of parasite isolates from regions of both prior to and after ART usage, but failed to identify potential mutations that might be indicative of directional ART-driven selection.

The MST network showed that the probable ancestral haplotype had a frequency of >50% and was present in all continents except South America. South America is distinctive in *pfatp6* polymorphisms (Figure S2C) and mostly likely reflects strongly structured parasite populations with high genetic differentiation [59]. The 3D7 haplotype was the second most prevalent and present in all continents. In addition, the star-like genealogy of the MST network suggests a past range expansion of the parasite populations as well as rapid evolution of the *pfatp6* gene, a scenario consistent with the evolution of highly specialized genes in *Plasmodium* [60]. However, studies conducted so far with *pfatp6* gene did not detect evidence of natural selection for the worldwide parasite populations and most evolution tests did not detect significant departure from neutrality [19], [22]. Specifically, the dN/dS values of <1 and neutrality index of McDonald-Kreitman test of >1 suggest that *pfatp6* is under purifying selection. Besides, the occurrence of all nonsynounymous mutations outside of the predicted ART-binding domain may suggest functional constraints of the pfatp6 protein. In this regard, the *pfatp6* polymorphisms are evolutionarily informative and have been used for calculating the most recent common ancestor [52] and estimating geographical expansion of *P. falciparum* populations from Africa [61]. Yet, it needs to be cautioned that evolution analysis using the McDonald-Kreitman test may have less power for populations with limited sample sizes [45], [62].

Parasite population structures are well mirrored from the estimates of *F*_ST_ values, which are used to differentiate populations or groups. *F*_ST_ is directly related to the degree of resemblance among individuals within populations, and to the variance in allele frequency among populations. Similar evolutionary processes can simultaneously increase the similarity among individuals within populations and differentiation among populations. Estimates of *F*_ST_ among 19 populations identified different levels of differentiation between populations from distinct geographic localities. The highest level of differentiation was between the Venezuela population and other populations from the rest of the world. Within the GMS, the two Thai populations collected prior to or after ART deployment showed only a low level of population differentiation, suggesting that ART use did not cause significant changes in parasite population structure in this region. It is interesting to notice that the *F*_ST_ values between China and other six GMS populations were near or over 0.15 (significantly different). China had a drug use history that is drastically different from other countries within the GMS, and ARTs have been applied to treating falciparum malaria for nearly 30 years [63]. Although it could be speculated that the population differentiation observed in China might be drug-driven or as the result of wide applications of ARTs, we could not identify SNPs that might be directionally selected by ARTs. The substructures observed among parasite populations may be due to differences in the local malaria epidemiology, which will greatly influence the estimates of genetic diversity of parasites if populations are pooled for analysis. Therefore, analysis aimed to identify genetic loci associated with drug resistance through genome-wide association studies needs to consider the genetic structure of the studied parasite populations.

The initial postulation of *pfatp6* as a prime target of ART family drugs based on heterologous expression and biochemical studies has spurred significant interests in validating mutations in this gene as molecular markers of ART resistance in *P. falciparum*. Sequencing of global *P. falciparum* field isolates has identified numerous mutations, most of which were population-specific and at low prevalence. The presence of abundant resistance-unrelated SNPs would make it difficult to identify resistance-associated mutations until they gain considerable prevalence. Some studies showed that deployment of ACTs were associated with changes in frequency of certain *pfatp6* mutations [25], [64]. To date, none of these mutations have been conclusively shown to be responsible for ART resistance. Since the GMS has the longest history of deployment of ARTs, *pfatp6* gene in parasites from this region in recent years would be expected to contain signature mutations designating ART resistance if *pfatp6* were indeed the primary target of ART drugs. However, mutations in *pfatp6* from the GMS parasite populations, albeit relatively abundant, were either infrequent or present also in other malaria endemic regions with more recent ART use histories. Besides, the clinical ART mutation detected in western Cambodia is not linked to mutations in *pfatp6* [8], [65]. Furthermore, in vitro selection experiments of ART resistance did not detect any changes at the *pfatp6* locus [66], [67]. Altogether, current available data challenge the suitability of *pfatp6* as a molecular marker of ART resistance in *P. falciparum*.

## Supporting Information

Figure S1 **A sliding window plot (window length 100 bp, step size 25 bp) of nucleotide diversity (π) of** ***pfatp6*** **gene in** ***P. falciparum*** **populations from the GMS.**
(PDF)

Figure S2 **Unrooted minimum spanning tree network showing genetic relationship among parasites from four continents.** A) Asia; B) Pacific Islands; C) South America; D) Africa.
(PDF)

Figure S3 **Unrooted minimum spanning tree network showing genetic relationship among** ***pfatp6*** **amino acid sequence for the worldwide** ***P. falciparum*** **populations.**
(PDF)

Figure S4 **Principal component analysis of the GMS parasite populations.** A) Screen plot indicating the proportion of variance accounted for in the first 3 eigenvectors or principal components (PC1, PC2, and PC3). B–D) Multidimensional scaling plots for each combination of PCs. Figures indicate the relative distances between each of the isolates, color coded by nation.
(PDF)

Table S1 **Statistics for** ***pfatp6*** **gene from the entire** ***falciparum*** **populations.**
(DOCX)

Table S2 **Genetic differentiation between continent-groups measured by** ***F*** **_ST_ (under the diagonal) and** ***Φ*** **_ST_ (above the diagonal) values.**
(DOCX)
