# Supplementary material for: Genetic Diversity and Lack of Artemisinin Selection Signature on the Plasmodium falciparum ATP6 in the Greater Mekong Subregion
Source: PLoS One. 2013 Mar 26;8(3):e59192. doi: 10.1371/journal.pone.0059192 (PMC3608609; doi:10.1371/journal.pone.0059192)
Supplement: Figure S1 — A sliding window plot (window length 100 bp, step size 25 bp) of nucleotide diversity (π) of pfatp6 gene in P. falciparum populations from the GMS. (PDF) [file pone.0059192.s001.pdf]

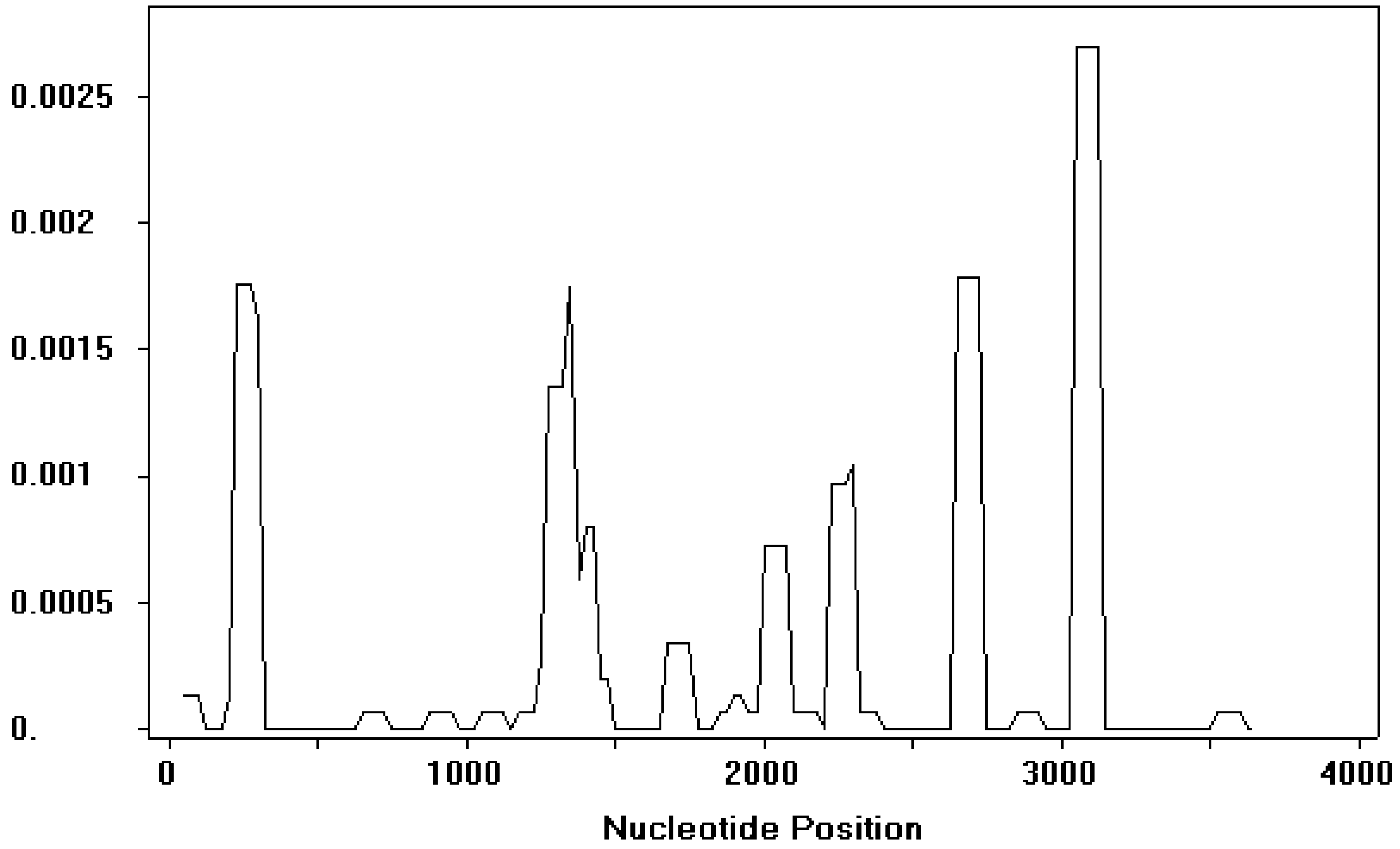

Figure S1. A sliding window plot (window length 100 bp, step size 25 bp) of nucleotide diversity (π) of *pfatp6* gene in *P. falciparum* populations from the GMS.
