# Supplementary material for: Genetic Diversity and Lack of Artemisinin Selection Signature on the Plasmodium falciparum ATP6 in the Greater Mekong Subregion
Source: PLoS One. 2013 Mar 26;8(3):e59192. doi: 10.1371/journal.pone.0059192 (PMC3608609; doi:10.1371/journal.pone.0059192)
Supplement: Figure S2 — Unrooted minimum spanning tree network showing genetic relationship among parasites from four continents. A) Asia; B) Pacific Islands; C) South America; D) Africa. (PDF) [file pone.0059192.s002.pdf]

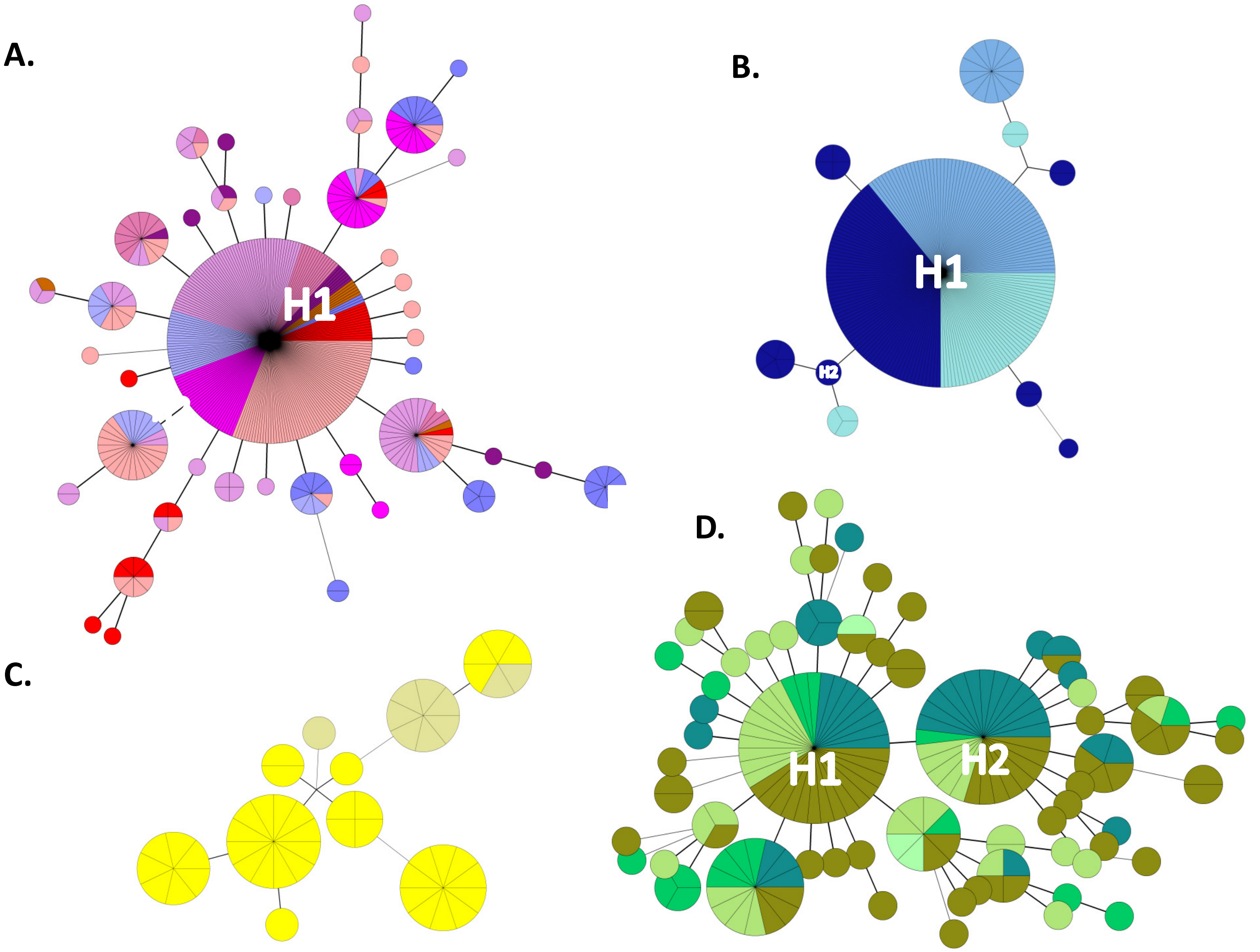

Figure S2. Unrooted minimum spanning tree network showing genetic relationship among parasites from four continents. A) Asia; B) Pacific Islands; C) South America; D) Africa.
