# Supplementary material for: Genetic Diversity and Lack of Artemisinin Selection Signature on the Plasmodium falciparum ATP6 in the Greater Mekong Subregion
Source: PLoS One. 2013 Mar 26;8(3):e59192. doi: 10.1371/journal.pone.0059192 (PMC3608609; doi:10.1371/journal.pone.0059192)

Figure S3. Unrooted minimum spanning tree network showing genetic relationship among *pfatp6* amino acid sequence for the worldwide *P. falciparum* populations.

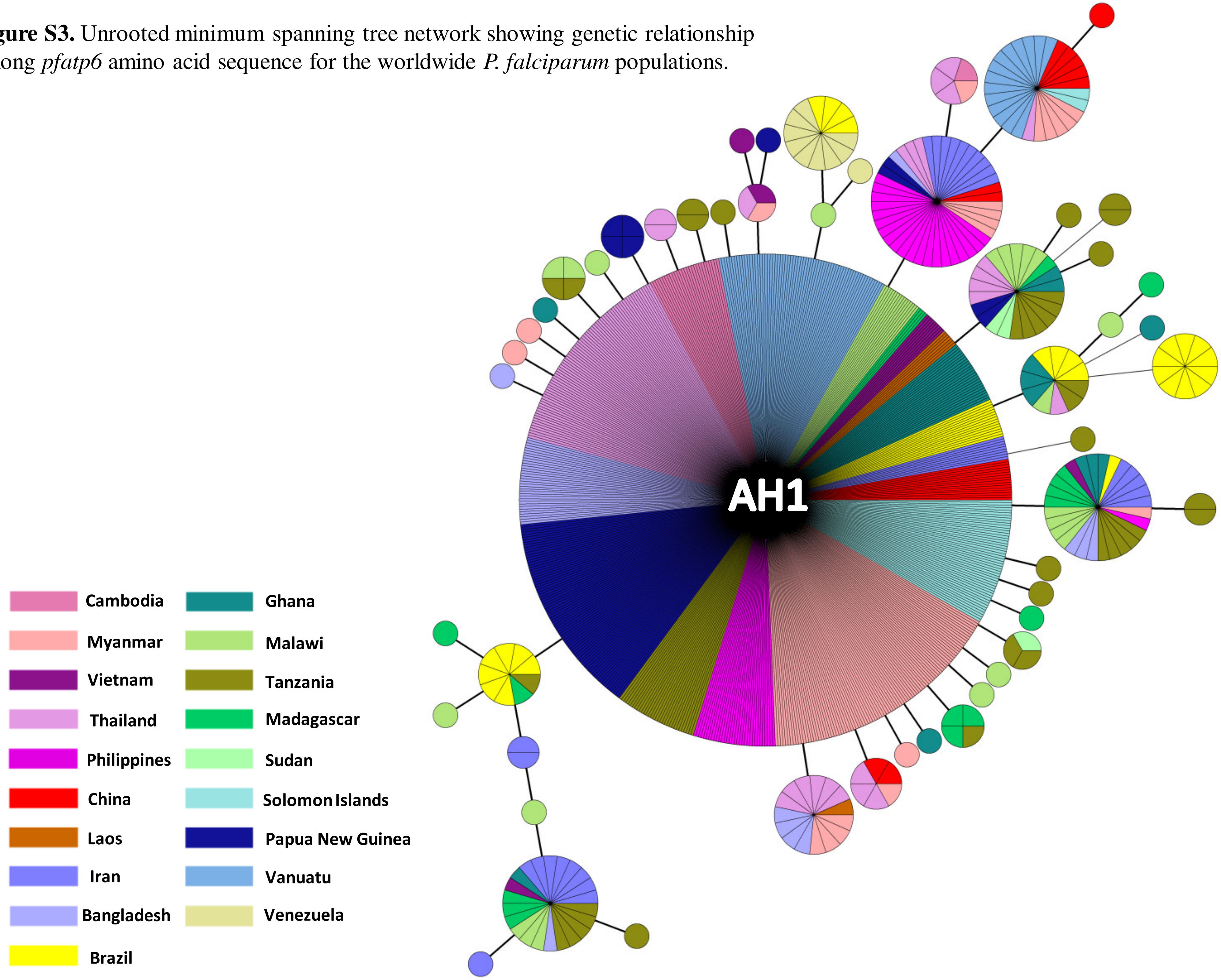

Supplement: Figure S3 — Unrooted minimum spanning tree network showing genetic relationship among pfatp6 amino acid sequence for the worldwide P. falciparum populations. (PDF) [file pone.0059192.s003.pdf]
