# Supplementary material for: Genetic Diversity and Lack of Artemisinin Selection Signature on the Plasmodium falciparum ATP6 in the Greater Mekong Subregion
Source: PLoS One. 2013 Mar 26;8(3):e59192. doi: 10.1371/journal.pone.0059192 (PMC3608609; doi:10.1371/journal.pone.0059192)
Supplement: Figure S4 — Principal component analysis of the GMS parasite populations. A) Screen plot indicating the proportion of variance accounted for in the first 3 eigenvectors or principal components (PC1, PC2, and PC3). B–D) Multidimensional scaling plots for each combination of PCs. Figures indicate the relative distances between each of the isolates, color coded by nation. (PDF) [file pone.0059192.s004.pdf]

A.

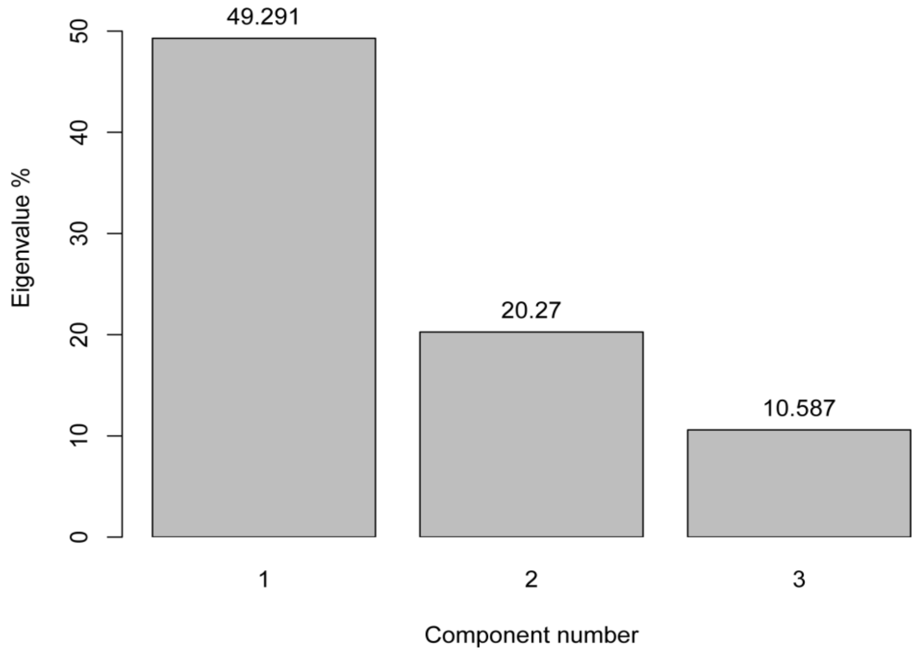

B.

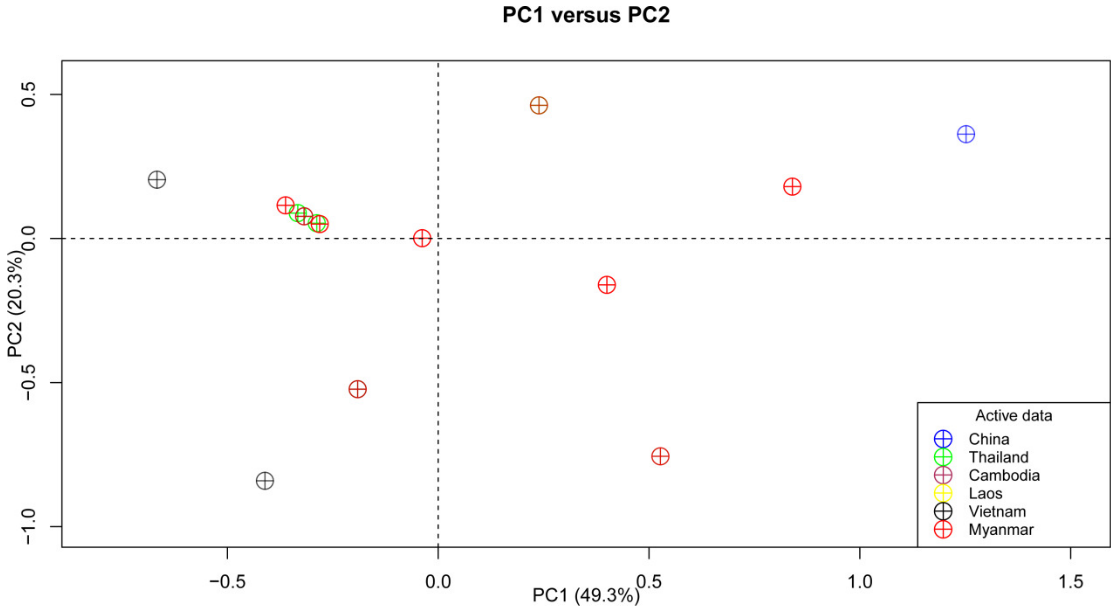

Figure S4. Principal component analysis of the GMS parasite populations. A) Screen plot indicating the proportion of variance accounted for in the first 3 eigenvectors or principal components (PC1, PC2, and PC3). B-D) Multidimensional scaling plots for each combination of PCs. Figures indicate the relative distances between each of the isolates, color coded by nation.

Figure S4

C.

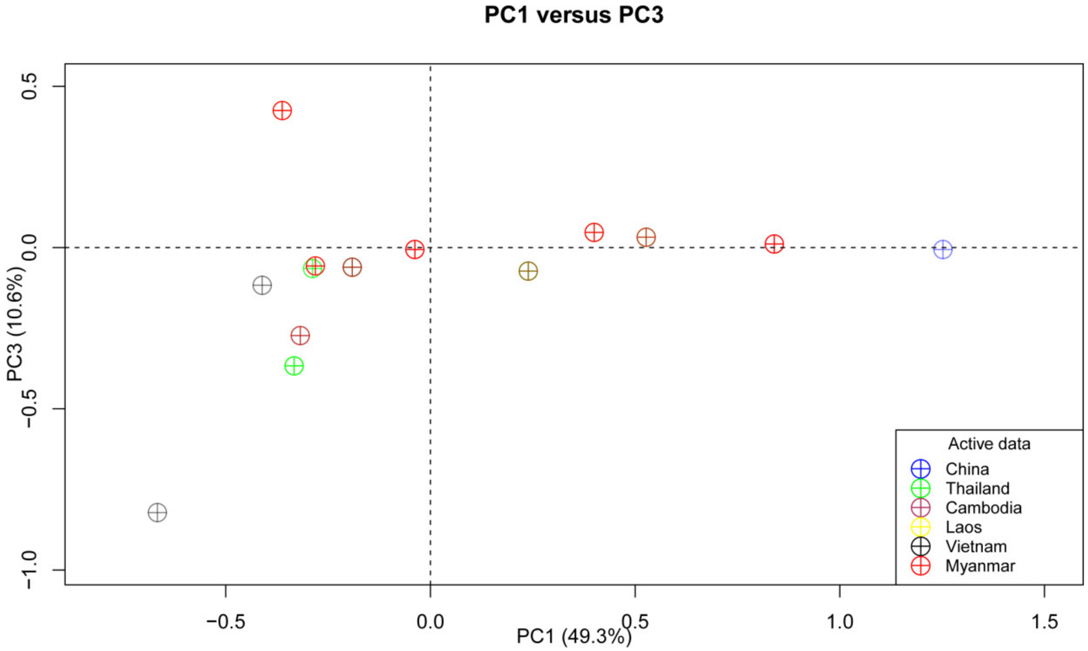
PC1 versus PC3
0.5
0.0
−0.5
−1.0
PC3 (10.6%)
−0.5
0.0
0.5
1.0
1.5
PC1 (49.3%)
Active data
China
Thailand
Cambodia
Laos
Vietnam
Myanmar

D.

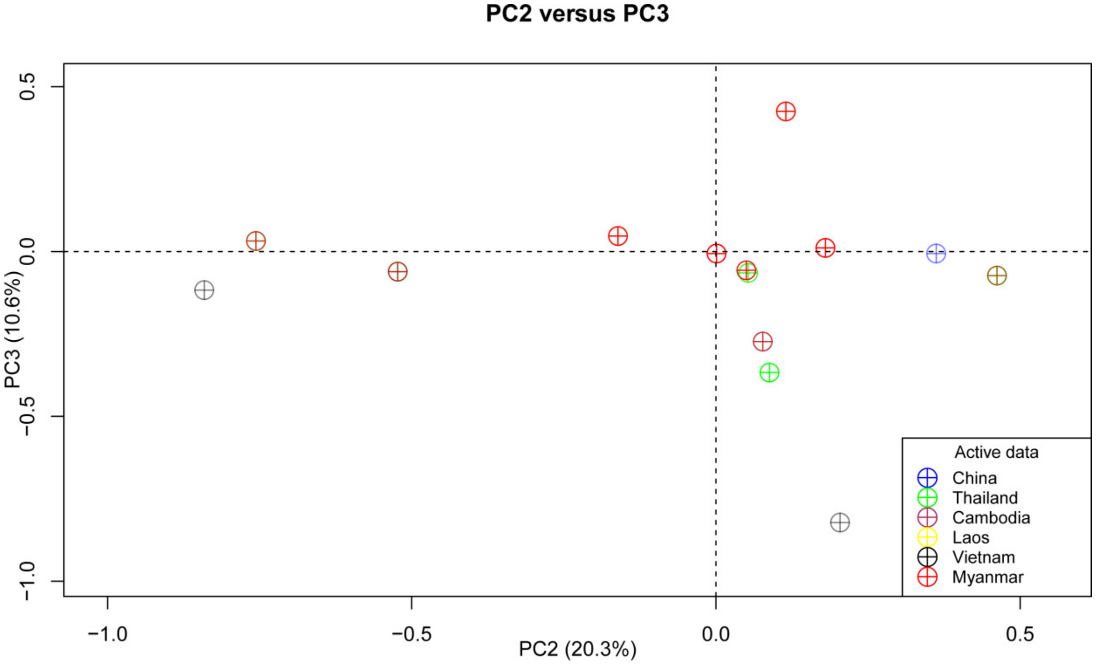
PC2 versus PC3
0.5
0.0
−0.5
−1.0
PC3 (10.6%)
−1.0
−0.5
0.0
0.5
PC2 (20.3%)
Active data
China
Thailand
Cambodia
Laos
Vietnam
Myanmar
