# Supplementary material for: Genetic Diversity and Lack of Artemisinin Selection Signature on the Plasmodium falciparum ATP6 in the Greater Mekong Subregion
Source: PLoS One. 2013 Mar 26;8(3):e59192. doi: 10.1371/journal.pone.0059192 (PMC3608609; doi:10.1371/journal.pone.0059192)
Supplement: Table S1 — Statistics for pfatp6 gene from the entire falciparum populations. (DOCX) [file pone.0059192.s005.docx]

**Table S1** Statistics for *pfatp6* gene from the entire *falciparum* populations. Several diversity indices were also indicated. *S*, segregating sites; *h*, haplotype diversity; *π*, nucleotide diversity; *k*, average number of pairwise differences. 1. This study. 2. From Tanabe et al. (2004)

| **Sample** | **Continent** | **Date of  collection** | **Samples  size** | **No. of  haplotypes** | **Unique haplotype** | ***S*** | ***h*** | ***π*** | ***k*** |
| --- | --- | --- | --- | --- | --- | --- | --- | --- | --- |
| **Asia** | **-** | **-** | **426** | **43** |  | **31** | **0.704±0.024** | **0.00034±0.00002** | **1.266** |
| China | Asia | 2005 | 26 | 8 | 3 | 6 | 0.695±0.091 | 0.00043±0.00007 | 1.588 |
| Laos | Asia | 2010 | 8 | 3 | 0 | 3 | 0.464±0.200 | 0.00020±0.00010 | 0.750 |
| Myanmar | Asia | 2007-2009 | 116 | 19 | 6 | 15 | 0.614±0.049 | 0.00027±0.00003 | 0.991 |
| Cambodia | Asia | after 2005 | 29 | 5 | 1 | 5 | 0.645±0.065 | 0.00023±0.00004 | 0.842 |
| Vietnam | Asia | 2006 | 13 | 7 | 2 | 7 | 0.731±0.133 | 0.00040±0.00011 | 1.462 |
| Thailand^1^ | Asia | 2007-2008 | 21 | 8 | 3 | 6 | 0.724±0.101 | 0.00030±0.00006 | 1.114 |
| Thailand^2^ | Asia | 1995 | 81 | 12 | 1 | 11 | 0.643±0.053 | 0.00028±0.00004 | 1.016 |
| Philippines | Asia | 1997 | 53 | 5 | 1 | 4 | 0.615±0.056 | 0.00024±0.00003 | 0.887 |
| Bangladesh | Asia | 2007 | 44 | 8 | 1 | 7 | 0.673±0.069 | 0.00024±0.00004 | 0.878 |
| Iran | Asia | 2001-2002 | 35 | 10 | 4 | 11 | 0.876±0.027 | 0.00070±0.00005 | 2.585 |
| **Pacific Islands** | **-** | **-** | **219** | **10** |  | **11** | **0.290±0.040** | **0.00018±0.00003** | **0.667** |
| PNG | Pacific Islands | 2001-2002 | 88 | 7 | 3 | 8 | 0.327±0.064 | 0.00015±0.00003 | 0.546 |
| Solomon Islands | Pacific Islands | 1996 | 51 | 3 | 2 | 4 | 0.185±0.071 | 0.00010±0.00004 | 0.380 |
| Vanuatu | Pacific Islands | 1997 | 80 | 2 | 0 | 3 | 0.292±0.055 | 0.00024±0.00005 | 0.877 |
| **Africa** | **-** | **-** | **166** | **62** |  | **43** | **0.922±0.013** | **0.00061±0.00003** | **2.240** |
| Ghana | Africa | 2004 | 37 | 13 | 6 | 13 | 0.830±0.046 | 0.00042±0.00006 | 1.550 |
| Sudan | Africa | 1993-1994 | 3 | 2 | 0 | 2 | 0.667±0.314 | 0.00036±0.00017 | 1.333 |
| Tanzania | Africa | 1993, 1998, 2003 | 69 | 36 | 26 | 30 | 0.942±0.018 | 0.00067±0.00005 | 2.480 |
| Malawi | Africa | 2000 | 38 | 19 | 11 | 14 | 0.919±0.029 | 0.00056±0.00006 | 2.060 |
| Madagascar | Africa | 2005 | 19 | 12 | 7 | 10 | 0.930±0.038 | 0.00074±0.00010 | 2.725 |
| **South America** | **-** | **-** | **51** | **10** | **5** | **10** | **0.863±0.021** | **0.00083±0.00005** | **3.046** |
| Brazil | S. America | 1985-1999, 2004-2005 | 41 | 8 | 2 | 9 | 0.823±0.031 | 0.00069±0.00006 | 2.559 |
| Venezuela | S. America | 1997 | 10 | 3 | 2 | 4 | 0.511±0.164 | 0.00026±0.00013 | 0.956 |
| Total | - | - | 862 | 106 | - | 71 | 0.725±0.017 | 0.00041±0.0002 | 1.528 |
