# Supplementary material for: Genetic Diversity and Lack of Artemisinin Selection Signature on the Plasmodium falciparum ATP6 in the Greater Mekong Subregion
Source: PLoS One. 2013 Mar 26;8(3):e59192. doi: 10.1371/journal.pone.0059192 (PMC3608609; doi:10.1371/journal.pone.0059192)
Supplement: Table S2 — Genetic differentiation between continent-groups measured by F ST (under the diagonal) and Φ ST (above the diagonal) values. (DOCX) [file pone.0059192.s006.docx]

**Table S2** Genetic differentiation between continent-groups measured by *F*_ST_ (under the diagonal) and *Φ*_ST_ (above the diagonal) values.

|  | **Asia** | **Pacific Islands** | **Africa** | **South America** |
| --- | --- | --- | --- | --- |
| **Asia** | - | 0.0193** | 0.155** | 0.5744** |
| **Pacific Islands** | 0.0171* | - | 0.2133** | 0.6631** |
| **Africa** | 0.0922* | 0.1371* | - | 0.5406** |
| **South America** | 0.2879* | 0.3792* | 0.1831* | - |

*, significant at *P*<0.05 by the permutation test; **, significant *P* values after Bonferroni correction.
